# Language impairment in the moderate stage of dementia due to Alzheimer's disease

**DOI:** 10.1590/0004-282X-ANP-2020-0123

**Published:** 2021-05-07

**Authors:** Karin Zazo ORTIZ, Juliana Onofre DE LIRA, Thais Soares Ciariancullo MINETT, Paulo Henrique Ferreira BERTOLUCCI

**Affiliations:** 1 Universidade Federal de São Paulo, Departamento de Fonoaudiologia, São Paulo SP, Brazil. Universidade Federal de São Paulo Universidade Federal de São Paulo Departamento de Fonoaudiologia São Paulo SP Brazil; 2 Universidade de Brasília, Ceilândia Campus, Brasília DF, Brazil. Universidade de Brasília Universidade de Brasília Ceilândia Campus, Brasília DF Brazil; 3 University of Cambridge, Department of Radiology, Cambridge, United Kingdom. University of Cambridge University of Cambridge Department of Radiology Cambridge United Kingdom; 4 Universidade Federal de São Paulo, Departamento de Neurologia e Neurocirurgia, São Paulo SP, Brazil. Universidade Federal de São Paulo Universidade Federal de São Paulo Departamento de Neurologia e Neurocirurgia São Paulo SP Brazil

**Keywords:** Dementia, Alzheimer Disease, Language, Demência, Doença de Alzheimer, Linguagem

## Abstract

**Background:**

During the moderate stage of dementia due to Alzheimer’s disease (AD), language disorder is more evident and it impacts on communication. An overview of language impairment could be helpful to find compensatory communication strategies for these patients.

**Objective:**

To identify all language impairments among patients with moderate-stage of AD.

**Methods:**

20 patients diagnosed with probable AD based on the criteria of the NINCDS-ARDRA, with a MMSE score of 13-23 points and CDR=2, who were undergoing treatment for AD with therapeutic doses of acetyl cholinesterase inhibitors, were assessed using the Boston Diagnostic Aphasia Examination (BDAE), a test that provides a broad assessment of language. The results were compared with the performance of a normal population.

**Results:**

The patients assessed in this study presented normal scores for oral and written word recognition, repetition, mechanics of writing, primer-level dictation and spelling to dictation but also had impairment at most levels of linguistic processing, in oral and written comprehension and production. In general, as expected, the tasks relying on access to the mental lexicon were most significantly affected. However, they performed well in the naming task, in which semantic cues were presented. Moreover, the patients assessed in this study had better performance in written comprehension tasks than in oral ones.

**Conclusion:**

The severity of the language impairments was not homogenous, with some linguistic abilities more impaired than others. The abilities that were found to be preserved can help to guide strategies for aiding in communication at this stage of AD.

## RESUMO

**Introdução:**

Durante o estágio moderado da demência na doença de Alzheimer (DA), o distúrbio de linguagem se intensifica, afetando a comunicação. A identificação minuciosa das habilidades linguísticas mais comprometidas nessa fase da doença pode auxiliar no uso de estratégias facilitadoras para a comunicação. Este estudo visou identificar as alterações de linguagem em pacientes com DA na fase moderada.

**Métodos:**

20 pacientes com o diagnóstico de DA provável com base nos critérios do NINCDS-ARDRA, MMSE entre 13 e 23 pontos, CDR=2 e em tratamento medicamentoso para DA foram submetidos à avaliação da global da linguagem por meio do teste de Boston para diagnóstico de Afasia. Os resultados obtidos foram comparados aos da população normal.

**Resultados:**

Os pacientes apresentaram escores normais nas tarefas de compreensão oral e escrita de palavras, repetição, mecânica da escrita, ditado do primeiro nível e soletração para o ditado, mas também apresentaram comprometimento em todos os níveis do processamento linguístico, tanto na compreensão quanto nas emissões oral e escrita. Em geral, como esperado, as tarefas envolvendo o acesso lexical foram mais afetadas, porém os pacientes obtiveram desempenho adequado para a nomeação responsiva em que a pista semântica é o eliciador para a resposta. Além disso, os pacientes tiveram melhor desempenho em tarefas de compreensão escrita do que oral.

**Conclusão:**

A gravidade da alteração linguística não foi homogênea com algumas habilidades linguísticas mais alteradas do que outras. As habilidades identificadas como preservadas podem contribuir para o uso de estratégias facilitadoras da comunicação nessa fase da DA.

## INTRODUCTION

Alzheimer’s disease (AD) is the most common cause of dementia, accounting for 50-75%, and is primarily a condition of later life, roughly doubling in prevalence every 5 years after age 65^1^. The commonest presentation of AD is in elderly individuals with insidious, progressive problems centered on episodic memory and difficulties with multitasking and loss of confidence. As the condition progresses, cognitive difficulties become more profound and widespread such that they interfere with activities of daily living^1^. The National Institute of Neurological Communicative Disorders and Stroke/Alzheimer’s Disease and Related Disorders Association (NINCDS/ADRDA) standard for the diagnosis of probable AD has previously defined that patients need to demonstrate memory changes and impairments in at least one of the following cognitive functions: language, attention, executive functions, visuospatial and psychomotor abilities^2^. While other cognitive skills have been well documented in this population, language deterioration warrants further research.

Concerns over language impairments in AD are not new. Many studies have sought to identify impairments at different levels of linguistic processing, like phonetic^3^, phonological^4^, lexical-semantic^5^ and syntactic^6^, along with deficits in discourse^7^. These studies have shown that language abilities become differentially impaired as the disease progresses. For example, phonetic and phonological processing are better preserved than lexical-semantic processing, in which changes are seen more frequently starting from the earliest stages in AD.

Clinically, it is during the moderate stage of the disease that communication difficulties are exacerbated. Numerous studies have shown a decline in cognitive domains with dementia stage^5^^,^^8^, although changes in the language abilities investigated seemed not to be homogenous. Nonetheless, despite the importance of language with regard to making differential diagnoses of neurodegenerative diseases, there is still a lack of language assessment tools in relation to dementia. More recently, some tests were proposed^9^, but there is still large use of tests that were developed to assess aphasia^10^, or use of screening tests.

Although tests developed to assess aphasia are not totally suitable for evaluating language deficits associated with neurodegenerative diseases^10^, they provide more data about language and cognition than do the tools normally applied for language screening among these patients. Moreover, it is worth bearing in mind that such tests need to undergo full psychometric analysis, including validity, internal consistency, reliability^11^ and other factors, and provide scores that take into consideration sociodemographic characteristics such as age and education, since these have an impact on language performance^12^.

Given the complexity of language, the impact of disease progression on each type of linguistic processing deserves be investigated in greater depth. Language deterioration should be monitored so that compensatory strategies aiding communication can be implemented. In such cases, an overview of language impairment could be helpful. With regard to AD, some controversies regarding language profile in relation to disease stage remain unresolved, with some studies considering that articulatory, phonological and syntactic abilities continue unscathed until the final stage of the disease^10^, while others consider that these also become compromised in some patients even in the early stages of the disease^3^^,^^4^^,^^5^^,^^6^^,^^7^.

Therefore, the objective of the present study was to identify language impairments among patients with dementia due to AD at the moderate stage of the disease.

## METHODS

A longitudinal study was conducted at the outpatient clinic of the Behavioral Neurology Division and at the Department of Speech, Language and Hearing Sciences of the Federal University of São Paulo. This study was approved by the local Research Ethics Committee (under no. 1606/03). Written informed consent was obtained from all enrolled subjects after they had received full information about the study.

### Subjects

The sample consisted of AD patients whose conditions were in accordance with the clinical criteria proposed by the NINCDS-ADRDA^2^ Work Group. The inclusion criteria were that the patients should not have any history of alcoholism or drug use; any use of psychotropic medications, except for atypical neuroleptics; or any visual or auditory impairments that could affect the outcome of the cognitive tests. The neurological assessment was performed by an AD expert. All of the patients diagnosed using these criteria underwent a complete neuropsychiatric evaluation followed by a neuropsychological evaluation. Cognitive screening tests, a neuropsychological battery and a functional assessment were used for patient selection and classification as presenting the moderate stage of AD. The Mini-Mental State Examination (MMSE) was used as a screening tool^13^. We used a Portuguese translation and Portuguese scoring of the MMSE^14^. Only individuals with an MMSE score greater than 12 and less than 24 points, who were undergoing treatment for AD with therapeutic doses of acetylcholinesterase inhibitors (donepezil ≥5 mg, rivastigmine ≥9 mg or galantamine ≥8 mg) were selected. The subjects were also assigned a clinical dementia rating (CDR)^15^. The CDR score was 2. For neuropsychological evaluation, the patients were assessed using the protocol established by the Consortium to Establish a Registry for Alzheimer’s Disease (CERAD)^16^, which addresses attention, memory, evocation, recognition, language, praxis, gnosia and abstract thinking using the following tests: verbal fluency, naming, word list memory, constructive praxis, word list evocation, word list recognition, apraxia evocation and the trail test.

All individuals who met the inclusion criteria were administered the Boston Diagnostic Aphasia Examination (BDAE)^17^. This test was chosen because it provides a broad assessment of auditory/oral and written comprehension and oral and written production. Only the tasks relating to language assessment were applied.

The following tasks were performed:

- Oral comprehension: word discrimination, body-part identification and complex ideational material.
- Speech tasks: automated sentences, repetition of words and repetition of high and low-probability sentences, oral reading of words and sentences, responsive naming, visual confrontation naming and animal fluency.
- Reading comprehension: symbol discrimination, word recognition, oral spelling, word-picture matching, comprehension of sentences and paragraphs.
- Writing: mechanics of writing, serial writing, primer level dictation, spelling to dictation, writing confrontation naming, narrative writing and sentences to dictation.

All patients were assessed by the same examiner. Individual assessments were performed in a quiet room and sessions lasted for less than one hour.

Based on the results from the assessments, analysis on the frequencies of the variables outlined above was conducted, together with comparative analysis of patient results versus suggested normative data^18^^,^^19^. We considering the impact of schooling on cognition, and that BDAE scores vary according to schooling. Therefore, in this study, in order to avoid that the results might be influenced by the demographic data, we made comparisons with scores published from normative data^18^^,^^19^. The scores from individuals with the lowest education (1-4 years of schooling) and from the oldest population were taken account for comparisons between AD patients and normal subjects.

### Statistical analysis

The chi-square test (without Yates correction) was used to compare categorical data. In situations in which contingency tables displayed expected values <5, Fisher’s exact test was applied.

Differences between the means of continuous data were tested using Student’s t tests for paired samples (t) (parametric), while the Wilcoxon Signed Rank (Z) test was used for the corresponding nonparametric samples. Parametric results were displayed when the two samples had similar results, while nonparametric results were shown when divergence occurred.

Bonferroni’s correction for multiple comparisons was used. P<0.002 was considered statistically significant and all tests were two-tailed. The Bonferroni correction compensates for an increase by testing each individual hypothesis at one significance level when there are many variables being tested. 95% confidence intervals (95%CI) were calculated for differences in means. Statistical analyses were carried out on a personal computer using the Statistical Package for the Social Sciences (SPSS) for Windows (version 11.5.1).

## RESULTS

### Sample characteristics

A total of 20 patients with moderate-stage AD were assessed. The patients’ ages ranged from 57 to 91 years, and their mean age was 75 years (±24.04 years). Their education level ranged from 2 to 11 years, with a mean of 4 years of formal study (±7.77 years). With regard to gender, 9 women and 11 men took part in the study. The patients had a mean score of 18 points (±6.36) in the MMSE.

The descriptive analysis on the AD patients’ results from the BDAE and the mean scores published for normal individuals is presented in Table 1.

The statistical analysis on the data comparing the performance of the two groups in the BDAE tasks is presented in Table 2.

**Table 1.** Descriptive analysis on Boston Diagnostic Aphasia Examination scores in Alzheimer’s disease patients and a normal population.

	Patients with moderate AD					Normal population (Radanovic and Mansur^19^)	
	Mean	SD	Median	Minimum	Maximum	Mean	SD
Oral comprehension							
Word discrimination	55.1	14.7	59.5	12.0	70.5	68.0	5.7
Body-part identification	15.3	2.5	16.5	9.0	18.0	18.9	1.3
Complex ideational material	6.3	1.3	6.0	4.0	8.0	9.5	2.0
Speech tasks							
Automated sequences	6.6	0.9	7.0	4.0	7.0	7.7	0.6
Repetition							
Words	9.2	1.3	10.0	6.0	10.0	9.8	0.4
High-probability phrases	6.5	1.8	7.0	2.0	8.0	7.5	1.2
Low-probability phrases	5.2	2.5	5.0	0.0	8.0	7.3	1.2
Naming							
Responsive	26.4	1.0	27.0	24.0	27.0	26.7	0.6
Visual confrontation	68.9	26.6	75.0	13.0	101.0	106.9	9.9
Animal fluency	8.6	3.5	8.0	4.0	15.0	18.0	7.0
Oral reading							
Words	22.6	7.5	24.0	7.0	30.0	26.9	2.6
Sentences	7.4	2.7	8.0	2.0	10.0	9.3	1.8
Reading comprehension							
Symbol discrimination	7.2	2.1	7.5	2.0	10.0	9.6	0.6
Word recognition	6.8	1.7	7.5	3.0	8.0	7.8	0.7
Oral spelling	2.2	2.1	1.0	0.0	6.0	5.1	2.7
Word-picture matching	6.1	3.1	7.0	0.0	10.0	9.2	2.0
Sentences and paragraphs	5.2	3.3	6.0	0.0	9.0	8.5	1.5
Writing							
Mechanics	3.4	1.6	4.0	1.0	5.0	4.7	0.7
Serial writing	31.6	10	30.5	8.0	45.0	43.0	5.5
Primer level dictation	11.1	2.9	12.0	2.0	14.0	13.6	2.4
Spelling to dictation	5.3	3.3	7.0	0.0	9.0	8.2	2.4
Written confrontation naming	3.8	3.3	3.0	0.0	9.0	8.9	2.3
Narrative writing	1.5	1.5	1.0	0.0	4.0	4.0	1.3
Sentences to dictation	4.7	4.0	5.0	0.0	10.0	10.8	2.7

AD: Alzheimer’s disease; SD: standard deviation.

**Table 2.** Comparison of mean Boston Diagnostic Aphasia Examination scores of Alzheimer’s disease patients and a normal population.

	Difference between means	T	Df	95%CI for difference	p-value
Oral comprehension					
Word discrimination	-12.9	-3.6	16	-20.5 to -5.4	0.002
Body-part identification	-3.6	-6.1	16	-4.9 to -2.3	<0.001*
Complex ideational material	-3.2	-9.8	15	-3.9 to -2.5	<0.001*
Speech tasks					
Automated sequences	-1.1	-5.0	16	-1.5 to -0.6	<0.001*
Repetition					
Words	-0.6	-1.9	16	-1.3 to 0.1	0.072
High-probability phrases	-1.0	-2.2	14	-2.0 to 0.0	0.044
Low-probability phrases	-2.1	-3.3	14	-3.5 to -0.7	0.005
Naming					
Responsive	-0.3	-1.4	15	-0.8 to 0.2	0.195
Visual confrontation	-38.0	-5.9	16	-51.7 to -24.3	<0.001*
Animal fluency	-9.4	-10.8	15	-11.3 to -7.6	<0.001*
Oral reading					
Words	-4.3	-2.3	16	-8.1 to -0.4	0.033
Sentences	-1.9	-2.9	15	-3.4 to -0.5	0.012
Reading comprehension					
Symbol discrimination	-2.4	-4.7	15	-3.5 to -1.3	<0.001*
Word recognition	-1.0	-2.3	15	-1.9 to -0.1	0.033
Oral spelling	-2.9	-5.1	12	-4.2 to -1.7	<0.001*
Word-picture matching	-3.1	-3.8	13	-4.9 to -1.3	0.002
Sentences and paragraphs	-3.3	-3.6	12	-5.4 to -1.3	0.004
Writing					
Mechanics	-1.3	-3.2	14	-2.2 to -0.4	0.007
Serial writing	-11.4	-4.2	13	-17.1 to -5.6	0.001*
Primer level dictation	-2.5	-3.3	14	-4.2 to -0.9	0.005
Spelling to dictation	-2.9	-3.3	13	-4.8 to -1.0	0.006
Written confrontation naming	-5.1	-5.5	12	-7.0 to -3.1	<0.001*
Narrative writing	-2.5	-6.1	12	-3.3 to -1.6	<0.001*
Sentences to dictation	-6.1	-5.5	12	-8.5 to -3.7	<0.001*

95%CI: 95% confidence interval.

## DISCUSSION

The most relevant finding from this study was that patients with moderate-stage ofAD had impairment at all levels of linguistic processing in oral and written comprehension and production. The severity of these impairments, however, was not homogenous for all different language processing. These findings will be discussed further in the ensuing text.

Regarding oral comprehension, the patients’ word discrimination was similar to that of healthy individuals. However, they had problems in the body-part identification task. Although the ability to carry out simple commands and recognize familiar stimuli appeared to be preserved among these AD patients, impaired body-part identification (which is deemed to be a simple stimulus) was evident. However, this task also involves items requiring identification of body side, thus rendering the stimulus more complex. The spatial orientation that is involved in this task has previously been reported as difficult for AD patients^20^.

The patients also encountered difficulty in the complex ideational material task assessing oral comprehension of complex sentences and texts. These difficulties had previously been reported even among mild AD patients^21^. It is thought that this may be associated with a decline in cognitive abilities, including working memory^22^, which allows temporary storage of linguistic information for information processing. This also enables the abstraction that is necessary for decoding and subsequently understanding sentences and small texts^21^.

Concerning speech tasks, automated sequences require recitation of hyperlearned verbal content through recruitment of the lexical-semantic system. The poor performance of AD patients relative to healthy subjects in this task was shown by the fewer items produced in each sequence. However, this constitutes an extremely simple task and so this difficulty in producing an automated series was unexpected. This suggests that marked lexical-semantic access problems exist at this stage of the disease, along with deficiencies in working memory for activation and production of items in serial order.

In general, tasks relying on access to the mental lexicon becomes significantly affected beginning in the early stages of AD^5^^,^^21^^,^^23^. In the visual confrontation naming task, patients should name the images, whereas in the animal fluency test they must provide animal names. Lower performance by normal individuals in these tasks suggests failures in lexical access or in the lexical buffer^23^, along with lexical-semantic impairment^24^^,^^25^. However, no differences were seen between moderate AD patients and healthy subjects in the naming task in which semantic cues were presented, i.e. the patients benefited from semantic cues indicating object function. This finding suggests that description of use/function improves lexical access for these patients.

In the word and sentence repetition tasks, the AD patients performed similarly to the normal individuals. By their nature, repetition tasks are simpler, because performing them does not rely on semantic-lexical association. The same holds for the reading aloud tasks. Indeed, reading aloud has been considered intact for “high-probability” stimuli, even when the reading comprehension ability appears to be affected.

For reading comprehension, the AD patients had poorer performance than healthy individuals, regarding symbol discrimination and word recognition. Symbol and word processing requires temporary storage of the information, to allow the pattern, letter style, font and shape corresponding to the code to be selected in the allographic buffer. This storage can be compromised, such that the correspondence between the letter shape presented and the corresponding shape fails or is disrupted. Impaired visual perception might also occur, thereby hampering the processing of essentially similar stimuli, which act as visual distractors and are naturally difficult for patients with visual-perceptual and attentional deficits^26^. This has an impact on reading processing.

The performance of the AD group in the word-picture matching task was similar to that of the normal group. The results showed that the oral and written word comprehension tasks remained intact for these patients.

Despite the difficulties in oral comprehension of sentences and paragraphs from the complex ideational material task outlined earlier, there was no performance difference between the AD patients and healthy individuals in the sentences and paragraphs reading task. Reading comprehension is a common complaint among AD patients early in the disease^27^^,^^28^. This ability relies on linguistic components and specific aspects of cognitive processing, such as memory^8^^,^^27^. The sentences and paragraphs reading subtest entails presentation of sentences and texts of different levels of complexity that require the patient to perform critical analysis of the content in order to select the correct answer. The AD patients’ performance was similar to that of normal individuals, particularly in relation to the shorter sentences, but was more impaired in the tasks involving longer texts. Overall, the patients assessed in this study had better performance in written comprehension tasks than in oral ones. This indicates that maintenance of the stimulus, in this case the written word, probably facilitated reprocessing of the information and its subsequent comprehension. In addition, the redundancy of the text^29^ and the multiple choice answers may also have aided comprehension.

In relation to writing, the performance of the patients was poorer than that of healthy individuals in serial writing, written confrontation naming, narrative writing and sentences to dictations tasks.

Writing impairments are common in AD patients^30^, although agraphia conditions are highly heterogeneous in the disease. This variation was evident in a study on mild and moderate AD groups, in which some patients had writing impairments, while others displayed normal performance in all tasks^21^.

The low performance of the patients in the serial writing task, in which patients should write alphabet and number sequences from 1 to 21, may have been due to failure in lexical access to the information in question. This difficulty might be attributable to a lexical-semantic disorder that also underlies language impairments and their relationships with numerical processing^31^. Alternatively, it might be attributable to memory deficit, where such that recall of the items in sequence is necessary in order to subsequently reproduce them.

The patients with AD had difficulties with written production. This low performance was expected among patients with moderate AD, since previous studies have shown writing impairments early on in the disease^32^. The involvement of semantic and lexical-semantic systems in these tasks may explain the performance seen in these cases, although these difficulties might also be related to impairments in the lexical-orthographic buffer^33^.

With regard to assessment of writing mechanics through examining motor aspects of handwriting, the patients with AD had similar performance to individuals without AD. Many authors have observed that patients with AD show progressive disorganization and degeneration of the various components of handwriting, such as the morphology of the letters^34^ and the graphic and spatial layout of letters and their arrangement in texts^35^. However, some heterogeneity and fluctuations have also been observed^36^.

 Individuals with and without AD had similar performance in the spelling to dictation and primer level dictation tasks, most probably because no semantic access or phonological lexicon is involved in this production. Although the patients with AD may have exhibited problems in writing longer words, these do not make up the majority of the stimuli, and therefore did not significantly impact the performance of these patients, regarding their scores in this subtask. In addition, writing might occur through a lexical or phonological route, in which preservation of the phonological route allows patients to match phonemes and graphemes, although this preservation does not necessary occur in all AD cases^37^.

As seen in different language aspects assessed through the BDAE test, many different language skills involving semantic factors and lexical access routes were impaired in the patient group, compared with the averages for the normal population. Language performance also depends on preservation of other cognitive factors that become impaired early on in AD and tend to worsen during the course of the disease. Naming abilities with semantic cues were preserved, especially with regard to object function. Reading comprehension among the patients assessed revealed a heterogeneous pattern of language deterioration. Maintenance of the stimulus, in this case the written word, probably facilitated reprocessing of the information and its subsequent comprehension among these patients with AD at the moderate stage of the disease.

 Thus, comprehensive language assessment furthers understanding of the language-cognitive abilities affected in these patients and helps to guide strategies for aiding communication, such as use of reading to facilitate oral comprehension and use of semantic cues to improve lexical access, among others, during the course of the disease.

This was a cross-sectional study. Although the language deterioration was heterogeneous, it is not possible to assume that this pattern would be the same for all patients. Although the control group did not have exactly the same demographic profile, we assume that all the differences found were related to AD, since we used the lowest scores obtained from the normal population, along with statistical correction. Further studies with larger populations are necessary, and all sociodemographic variables need to be controlled for, in order to confirm whether there is any pattern for language disorder in the moderate stage of AD.
